# Cold adaptive potential of pine wood nematodes overwintering in plant hosts

**DOI:** 10.1242/bio.041616

**Published:** 2019-04-25

**Authors:** Zhenkai Liu, Yongxia Li, Long Pan, Fanli Meng, Xingyao Zhang

**Affiliations:** 1 Laboratory of Forestry Pathogen Integrated Biology, Research Institute of Forestry New Technology, Chinese Academy of Forestry, Beijing, 100091, People's Republic of China; 2 Co-Innovation Center for Sustainable Forestry in Southern China, Nanjing Forestry University, Nanjing Jiangsu, 210037, People's Republic of China

**Keywords:** Pine wood nematode, Overwintering, Fatty acid composition, Cryoprotectant

## Abstract

The pine wood nematode (PWN; *Bursaphelenchus xylophilus*) is the causal agent of pine wilt disease, which results in severe ecological and economic losses in coniferous forests. During overwintering, PWNs undergo morphological and physiological changes to adapt to low temperature environments. Here, the physiological changes of the PWN populations sampled in the summer and winter were compared to analyze the role of low temperatures in their response. The PWN overwinters as third-stage dispersal juveniles, which showed significantly greater survival rates than summer populations (propagative forms) at sub-zero temperatures. The major biochemical compounds in the populations were analyzed by gas chromatography. Eight dominant fatty acids, with stearic acid being the most important, were identified from PWN propagative stage and third-stage dispersal stage. Compared with the propagative stage, the dispersal stage showed significant increases in the fatty acid content and the proportion of unsaturated fatty acids. Three carbohydrates, trehalose, glycerol and glucose, were detected in the PWN. Compared with the summer population, the levels of trehalose and glycerol increased significantly, while glucose decreased, in the winter population. The modifications in fatty acid composition and cryoprotectant levels, as elements of its changing physiology, play important roles in the overwintering success of the PWN.

## Abstract (precis)

**Summary:** The modifications in fatty acid composition and cryoprotectant levels, as elements of its changing physiology, play important roles in the overwintering success of the pine wood nematode.

## INTRODUCTION

The pine wood nematode (PWN), *Bursaphelenchus xylophilus* (Nematode: Aphelenchoididae), is the causal agent of pine wilt disease (PWD), which causes severe ecological and economic losses in coniferous forests (Mota and Vieira, 2008). The PWN is an invasive species introduced from North America that is causing extensive damage to pine trees in Asia, especially in China and Japan. In 1982, a PWN infection was first reported in Nanjing (N 32°03′, E 118°50′), subtropical China (Yang et al., 2003). By 2007, PWN infections had been found in 113 Chinese counties of 12 provinces, with the coastal regions in the subtropical and warm-temperate zones of China (N 29°–34°), including Zhejiang and Jiangsu Provinces, being the most seriously affected (Yu et al., 2011). By 2018, PWN infections had been found in 315 Chinese counties of 16 provinces, with the northeast regions in the mid-temperate zone of China (N 41°, E 124°), including Liaoning Provinces, being seriously affected (Provinces of Liaoning) (State Forestry Administration of the People's Republic of China, 2018). As a potential habitat, the PWN may infest more areas in Northeastern China and expand further into high-latitude cold regions.

The ability to survive cold is an important factor in determining the northern range limits of nematodes, but little is known about the PWN's cold tolerance. To survive the cold season, many nematodes display increasing levels of cold hardiness, which are regulated by antifreeze proteins and polyols as well as other low-molecular-weight cryoprotectants, such as amino acids and sugars (Smith et al., 2008; Wharton et al., 2003; Wu et al., 2018). Compared with other nematodes, the PWN has two developmental forms in its life cycle: propagative and dispersal. In the summer, individuals in the propagative phase reproduce rapidly, developing from eggs through four larval stages (J_1_–J_4_) to the reproductive adult, which increases the number of nematodes in infected pines and causes severe damage to healthy pines. In the autumn, with increasingly low temperature and food scarcity, the PWN molt from J_2_ into third-stage dispersal juveniles (J_III_). J_III_ molt to fourth-stage dispersal juveniles (J_IV_) when the juvenile receives a chemical signal released by the last instar larvae and pupae of the *Monochamus* beetle (Zhao 2et al., 2007; Pereira et al., 2013). The J_III_ is considered environmentally resistant to low temperatures and desiccation and is the stage that survives and disperses in the winter (Kondo and Ishibashi, 1978; Zhao et al., 2007).

Low temperatures bring several challenges to PWN survival. Some challenges are associated with low temperature per se, which may cause changes in the viscosity, phase and organization of membranes, with a corresponding loss of function (Wharton and Perry, 2011). Extreme sub-zero temperatures kill nematodes by freezing their cell contents and body water, and it is usually ice crystal formation inside nematodes that irrevocably damages its cells and body structures, eventually leading to death. To cope with these cold injuries, several physiological and biochemical mechanisms have evolved in overwintering nematodes. Physiological processes during this time are prioritized towards surviving the extreme conditions and conserving resources for the subsequent growing season, including the accumulation of lipid droplets and sugars (Sinclair, 2015; Wharton et al., 2000; Jagdale and Gordon, 1997).

Lipids are important components of nematode overwintering energetics, and play important roles in structuring water and ice during overwintering. Overwintering individuals, such as *Steinernema feltiae* and other insects, have significantly greater ratios of unsaturated to saturated fatty acids (Sinclair and Marshall, 2018; Jagdale and Gordon, 1997). Unsaturated fatty acids with low melting points probably allow lipid membranes to maintain the sufficient fluidity required to maintain protein functions at low ambient temperatures (Nieminen et al., 2013; Vukašinović et al., 2015). The accumulations of low-molecular-weight sugars and polyhydric alcohols (polyols) are well-known responses to freezing, drought, salt and osmotic stresses in the entomopathogenic nematodes *S.* *feltiae* and Antarctic nematodes *Panagrolaimus davidi* (Ali and Wharton, 2015; Wharton and Perry, 2011). In *S. feltiae* and other insects, trehalose and glycerol may accumulate as cryoprotectant agents that are correlated with survival at low temperatures (Khani et al., 2007; Ali and Wharton, 2015).

The aim of the present study was to describe the physiological and biochemical adaptations of the PWN to low temperature. The winter populations, or overwinter states, were generally more resistant to low temperature-associated stress than other populations. Therefore, the major physiological substances were measured by gas chromatography in field-collected summer and winter populations, to determine which factors are associated with the winter survival of the PWN.

## RESULTS

### Morphological characteristics

**Fig. 1.**
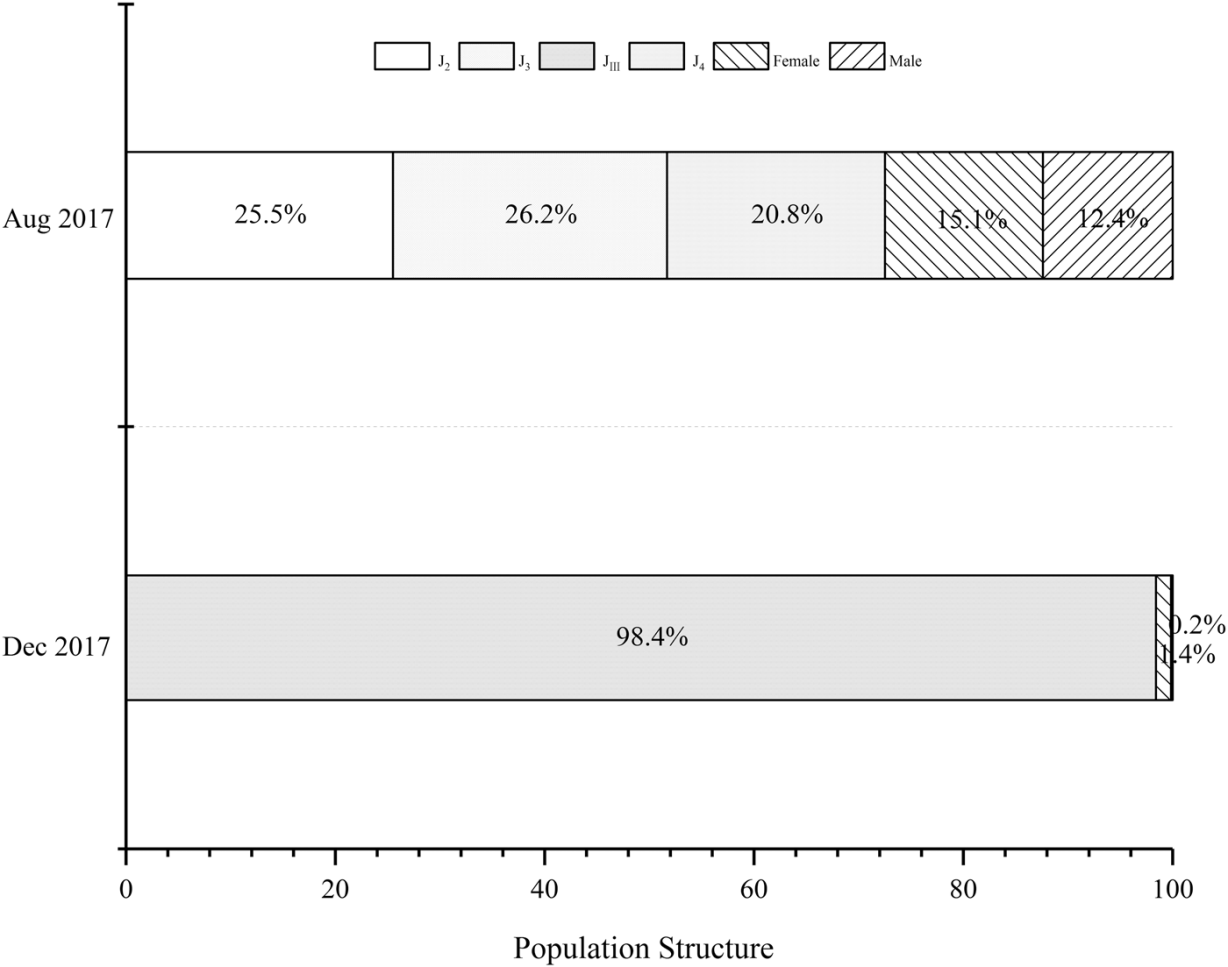
**Summer and winter pine wood nematode population structures.** The summer population (August 2017) included 2nd instar juveniles (J_2_), 3rd instar juveniles (J_3_), 4th instar juveniles (J_4_), females and males. The winter population (December 2017) included third-stage dispersal juveniles (J_III_), females and males.

The nematodes isolated from the infected wood collected in August 2017 were propagative forms of the PWN, including larval stages (J_2_–J_4_), and reproductive adults. The nematodes isolated in December 2017 were almost all J_III_ of the PWN (Fig. 1).

**Fig. 2.**
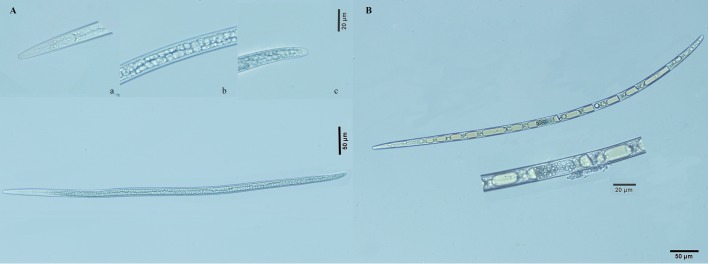
**Morphological characteristics of the third-stage dispersal juveniles of the pine wood nematode.** (A) Third-stage dispersal juveniles; (a) stylet and median bulb; (b) lipid droplets; (c) broadly rounded tail; (B) internal ‘gas bubbles’.

Morphologically, J_III_ is not clearly different from propagative larvae; both having a stylet and median bulb; however, the former has a broadly rounded tail and a deep deposition of lipid droplets in the intestine. Under an optical microscope, the PWN were dark but the tails were bright (Fig. 2A). Internally, the large refringent bodies were observed in some samples (Fig. 2B). The lipid droplets within the nematodes were stained red (Fig. S1). The intensity and precision of the lipid staining was greater in the dispersal larvae than the propagative larvae.

### Freezing temperature survival rate

**Fig. 3.**
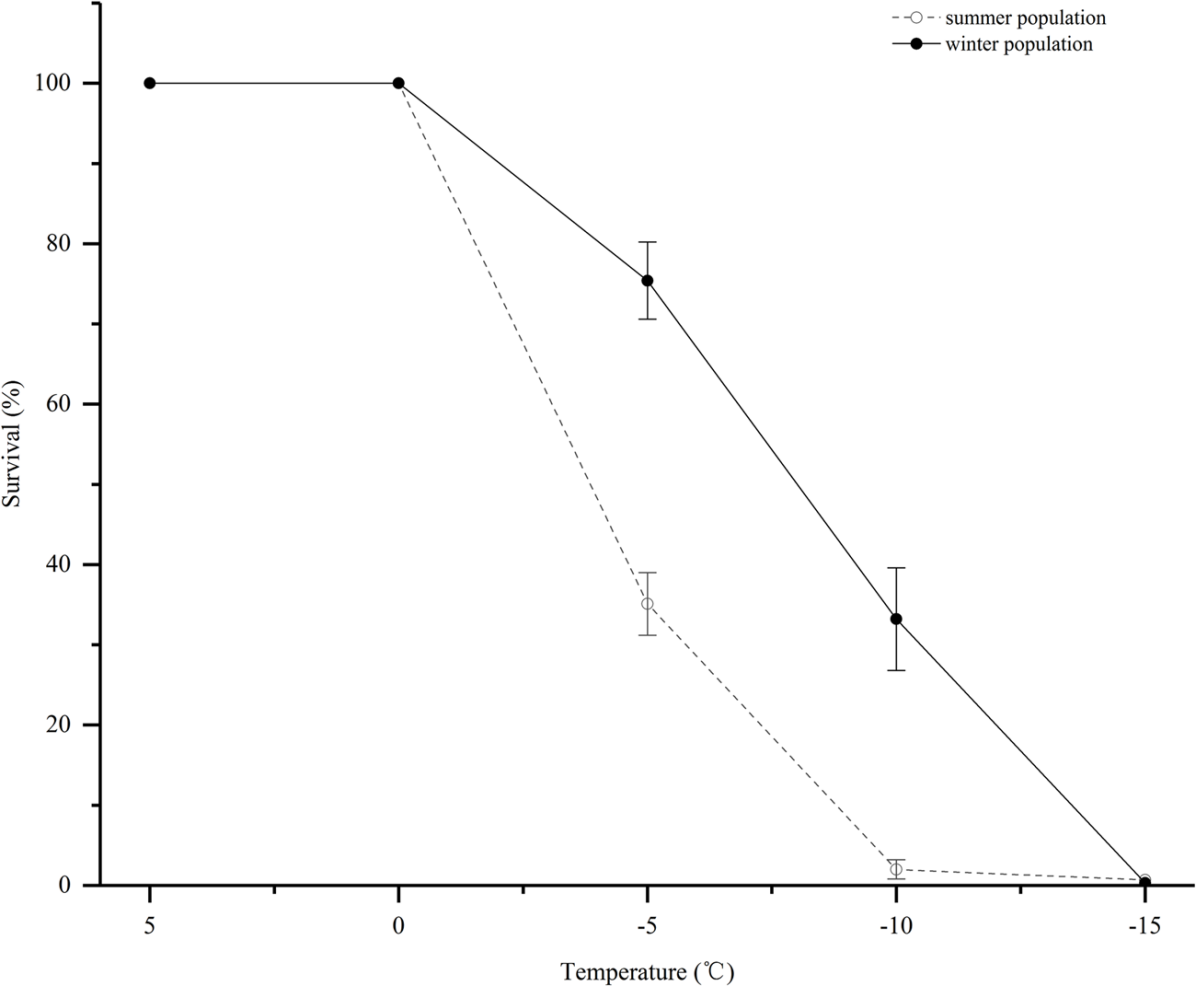
**Effects of temperature on the survival of the pine wood nematode.** Open circles, summer population; closed circles, winter population. Vertical bars represent standard errors.

**Fig. 4.**
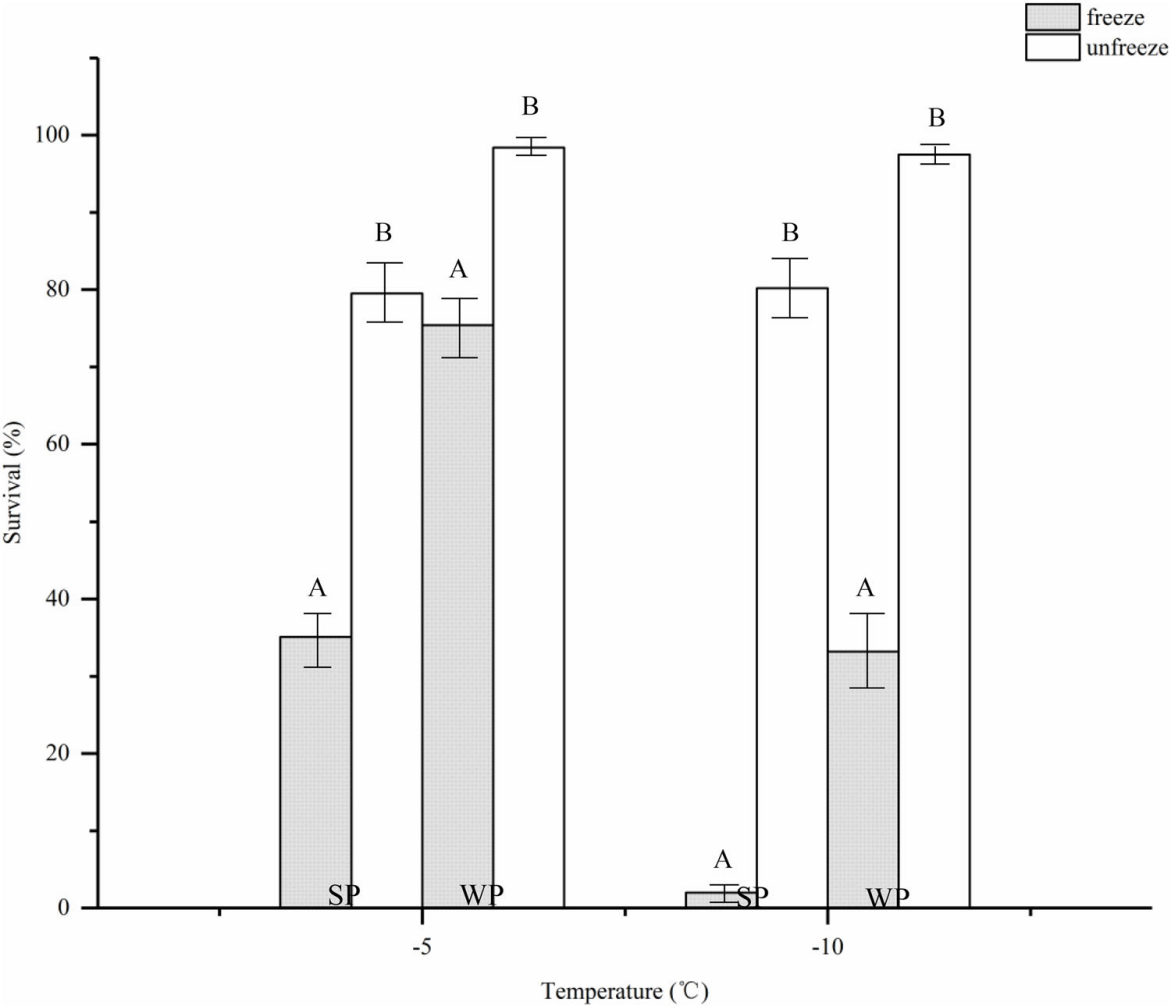
**Effects of supercooled water on the survival of the pine wood nematode.** SP, summer population; WP, winter population. Vertical bars represent standard errors.

No mortality was observed at temperatures greater than 0°C. The effects of sub-zero temperatures on PWN's survival in the freezing regime was significant. As the temperature decreased, the survival rate also decreased significantly (Fig. 3). Between 0°C and −15°C, the survival rate of J_III_ after the medium was frozen was notably greater than that of the propagative forms. Additionally, the S_50_ of the J_III_ was −8.2°C, which was lower than the −4.2°C of the propagative forms. When below −15°C, all the PWN died. The nematode suspensions could be kept in liquid phases at −5°C and −10°C when the suspensions were sufficiently clean and no ice crystals were added. Under these conditions, there were no significant differences between the survival rates of the propagated PWN at −5°C and −10°C. The same was true for the dispersal forms. However, when the temperature was below −15°C, the nematode suspension froze. Compared with frozen nematode suspensions, the nematode survival rates were significantly greater when the nematode suspension did not freeze (Fig. 4). At −5°C and −10°C the survival rates of propagative forms increased 2.26 times and 40.1 times, and those of J_III_ increased 1.3 times and 2.94 times, respectively. The lower the temperature, the greater the damage to the PWN caused by freezing the suspensions.

### Fatty acid profile

**Fig. 5.**
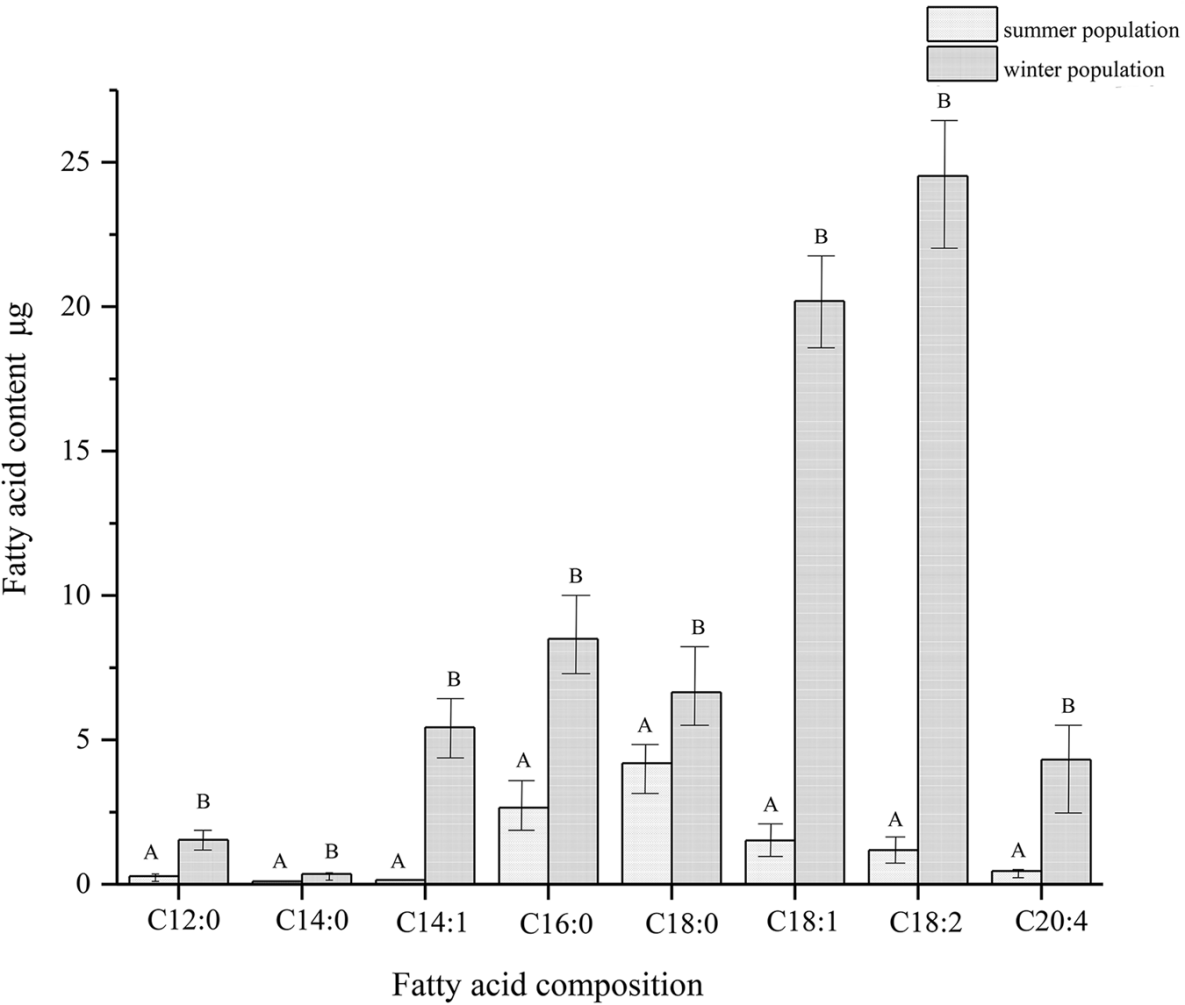
**Fatty acid compositions of pine wood nematode populations.** Using 2500 nematodes in a sample.

**Fig. 6.**
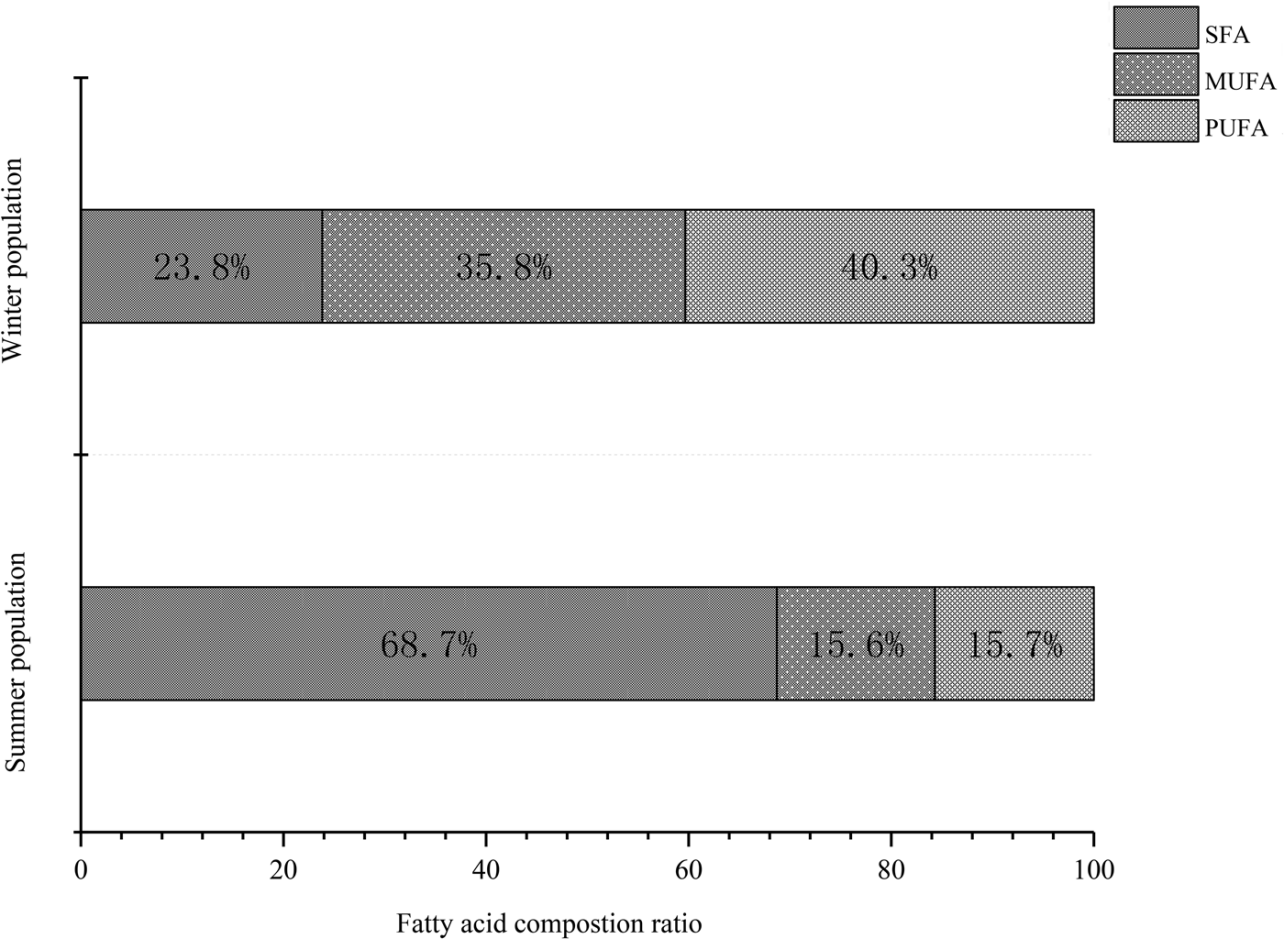
**Fatty acid composition ratios of pine wood nematode populations.** SFA, saturated fatty acids; MUFA, monounsaturated fatty acids; PUFA, polyunsaturated fatty acids.

Lipids comprised, on average 26.3% and 70.1% of the PWN dry weights at the propagative stage and third-stage dispersal, respectively, and the average propagative PWN dry weight was 0.028 μg/ind, while that of the dispersal was 0.041 μg/ind. Eight dominant fatty acids were identified from PWN propagative stage and third-stage dispersal individuals, including four kinds of saturated fatty acids, lauric acid (C12:0), myristic acid (C14:0), palmitic acid (C16:0) and stearic acid (C18:0), and four kinds of unsaturated fatty acids, myristoleic acid (C14:1), oleic acid (C18:1), linoleic acid (C18:2) and arachidonic acid (C20:4) (Fig. 5). The dispersal and propagative forms of PWN contained identical fatty acid components, but the relative quantities of the individual acids often varied. The total fatty acid content of the dispersal forms was 3.8 times that of the propagative forms. The propagative forms contained mainly saturated fatty acids (68.7%), of which C18:0 (39.9%) and C16:0 (25.2%) were the two dominant fatty acids. However, the dispersal forms contained mainly unsaturated fatty acids (76.2%), with C18:2 (35.3%) and C18:1 (28.2%) being the two dominant fatty acids. During the conversion of the PWN from the propagative to dispersal stages, the unsaturated fatty acid content increased significantly more than the saturated fatty acids, with oleic acid C18:1 increasing by 12.3 times, linoleic acid C18:2 increasing by 19.7 times, palmitic acid C16:0 increasing by 2.2 times and stearic acid C18:0 increasing by 0.58 times. The accumulation of unsaturated fatty acids leads to an increased ratio of unsaturated fatty acids to saturated fatty acids during the transition from the reproductive to dispersal type of PWN (Fig. 6). The proportions of C18:0, C18:1 and C18:2 was changed from 3.5: 1.3: 1 to 1: 3.0: 3.8. The unsaturated fatty acids increased from 32.3% to 76.1%.

### Sugars and polyols

**Fig. 7.**
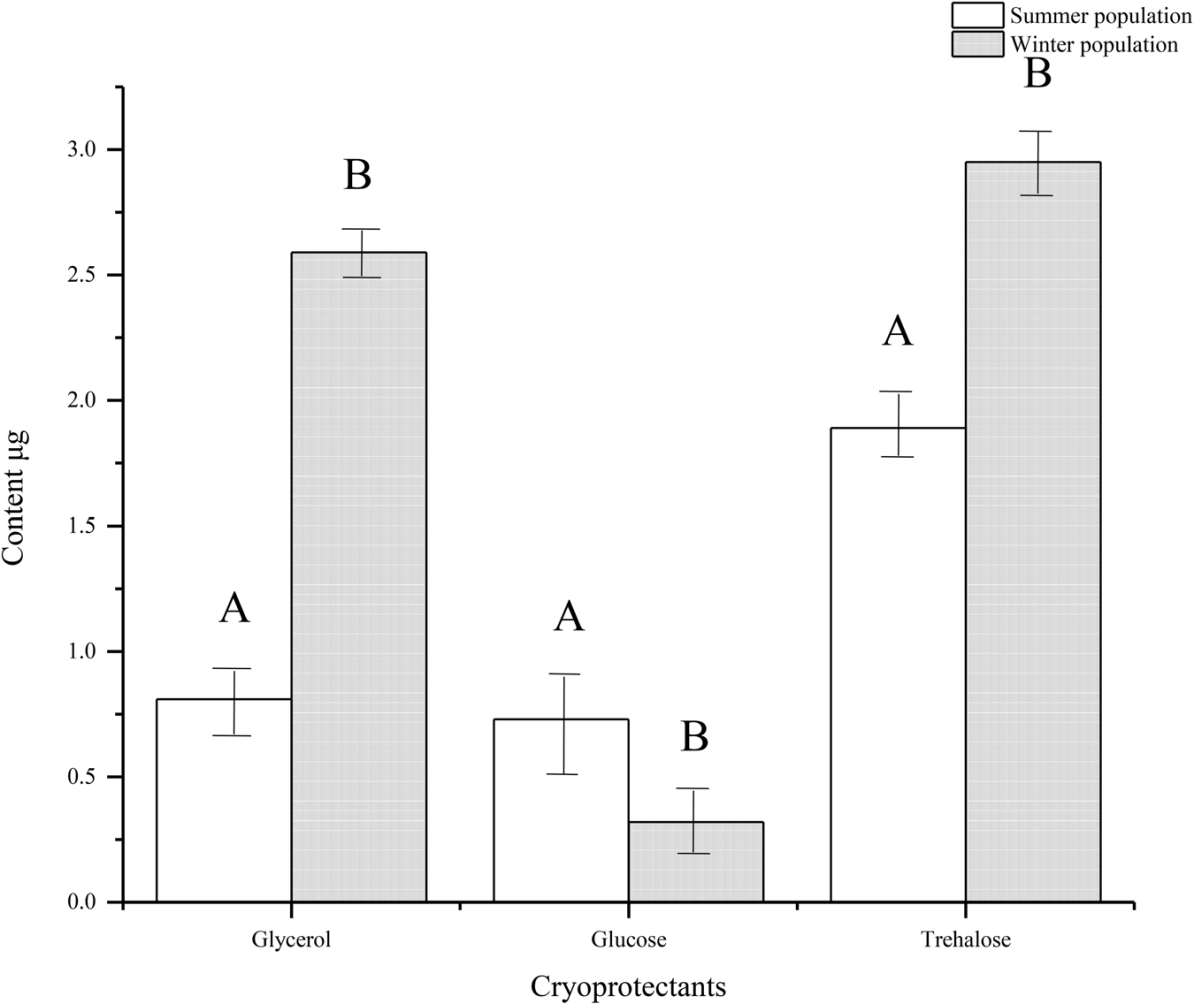
**Types of carbohydrates in pine wood nematodes.** Using 5000 nematodes in a sample.

Three carbohydrates, trehalose, glycerol and glucose, were detected in the PWN propagative and dispersal stages (Fig. 7). The glycerol, glucose and trehalose contents in the propagative stage were 0.81, 0.73 and 1.89 μg, respectively, and 2.59, 0.32 and 2.95 μg, respectively, in the dispersal stage. The glycerol and trehalose contents in the dispersal stage were greater than in the propagative type, and the glucose content was lower than in the reproductive type. During the conversion of the PWN from the propagative to dispersal stage, the glycerol and trehalose concentration increased significantly (*P*<0.05), with glycerol increasing by 220% and trehalose increasing by 56%. However, the glucose concentration significantly decreased (*P*<0.05) by 56.2%.

## DISCUSSION

To overcome low temperature-associated stresses in winter, the PWN molt to a particular developmental stage (J_III_), which corresponds to the pre-dauer (J2) stages of *C. elegans*, but they can sustain the diapause stage for a long time (Zhao et al., 2007). As with other diapause or dauer nematodes, the J_III_ are considered to be more resistant to cold than other stages. In some J_III_, the large refringent bodies became apparent, giving them a beaded appearance (Fig. 2B). In *Ditylenchus dipsaci*, the large refringent bodies were formed by lipid droplets that coalesced in the rehydration process (Wharton et al., 1985). In the PWN, the large refringent bodies may be formed by the encapsulation of many lipid droplets in the rehydration process (Fig. 2B).

The low temperature survival rate was used to measure the cold tolerance of nematode. Survival rates at −5°C and −10°C suggested that PWN's have a strong cold tolerance. In freezing water, the nematodes were seeded by exogenous ice nucleation and a proportion were freeze tolerant. However, in supercool water the nematode survival rate significantly increased. These results indicate that the PWN was freeze tolerant when exposed to frozen surroundings, but cold intolerance was enhanced when the PWN was free of exogenous ice nucleation and able to supercool (Raymond and Wharton, 2016). Survival rates for summer and winter populations indicated that the dispersal stage was more resistant to low, and especially freezing, temperatures than the propagative stage. Nematode suspensions cannot be kept unfrozen at temperature less than −15°C (Huang et al., 2018). Therefore, the effects of whether the medium freezing on PWN survival rates were not analyzed below −15°C.

The J_III_ stage is considered environmentally resistant to freezing temperatures because it contains densely packed lipid droplets (Zhao et al., 2007). Like other nematodes and insects, the fatty acids of the PWN are mainly composed of palmitic acid, stearic acid, oleic acid and linoleic acid, but palmitoleic acid (C16:1) was not detected (Jagdale and Gordon, 1997; Vukašinović et al., 2015). The difference in the fatty acid profiles may be related to species and environmental factors (Thompson, 1973).

Compared with the propagative stage, the dispersal stage had significant increases in the fatty acid content and the proportion of unsaturated fatty acids, which provide an energy base for PWNs and protect the fluidity of the cell lipid membrane to resist solidification at low temperatures (Murray et al., 2007). Stearic acid was the most important fatty acid in PWN. In propagative forms, stearic acid accounted for 40% of the fatty acid content. In dispersal forms, stearic acid was consumed to produce increased levels of oleic and linoleic acids. This caused the proportions of C18:0, C18:1 and C18:2 to change from 3.5: 1.3: 1 to 1: 3.0: 3.8. If the fatty acids of the PWN are self-synthesized, then there are a variety of desaturases. Δ9-desaturas transforms saturated fatty acid (C18:0) into monounsaturated fatty acid oleic acid (C18:1), and Δ12-desaturase transforms 18:1 into 18:2 (Nieminen et al., 2013). Lipids, as an energy store, are required for metabolic fuel over the winter and for the energetic demands of development in the spring (Sinclair and Marshall, 2018). The modifications to the fatty acids can contribute to temperature adaptation by allowing lipid membranes to maintain their fluidity at low ambient temperatures (Lyons et al., 1975; Vukašinović et al., 2015; Zhao et al., 2007).

Cryoprotectants, such as sugars and polyhydric alcohols, protect organisms from chilling and freezing injury and thereby enhance their cold tolerance (Wharton et al., 2000). However, the cold treatment conditions and seasonal changes determine the types of cryoprotectants in nematodes (Ali and Wharton, 2015; Grewal and Jagdale, 2002; Jagdale and Grewal, 2003). Trehalose, glycerol and glucose were the principal carbohydrates detected in the PWN. Compared with the propagative type, the glycerin and trehalose content was greater in the dispersal stage of PWN, while the glucose content was lower.

In the summer and winter populations of the PWN, the principal carbohydrate that had the highest content was glycerol, which is a penetrating cryoprotectant and readily permeates across membranes, playing an important role in freezing-avoiding insects (Qiu and Bedding, 2002). Therefore, its role during freezing could be more important than that of trehalose. Among non-penetrating cryoprotectants, trehalose had the greatest content in the PWN. Trehalose stabilizes membranes and protects against the dehydrating effects of osmotic stresses under low temperature stress (Wharton et al., 2000). The decrease in glucose may result from its use as a fuel for the basal metabolism during the overwintering period (Kimur et al., 1992).

Under laboratory conditions, the mortality rate of the PWN at −15°C was 100%. However, it can overwinter in northern China, where the lowest winter temperature can reach −30°C, the monthly minimum temperature is −21.6°C, and the monthly average temperature is −14.3°C (January 2018, Fushun). This illustrates that the PWN is able to survive low temperatures in the field, where the hosts play important roles in the overwintering of the PWN. The PWN spends the winter months in the host, which have minimum temperatures that are higher than the air temperature. According to the model established by Vermunt et al. (2012), the mean daily under-bark minimal temperature was, on average, ∼2.1°C higher than the minimum air temperature (Vermunt et al., 2012). The injuries to PWNs caused by low temperature were mainly the result of inoculative freezing. In winter, PWNs are distributed in the resin canals. The free water content in the host is reduced and xylem parenchyma cells adapt to sub-freezing temperatures by deep supercooling (Kasuga et al., 2006). This reduces the chance that the PWN will contact free water, thereby avoiding the influence of inoculative freezing. Therefore, the cold tolerance of plant-parasitic nematodes is a complex system, and the mechanisms behind the overwintering of the PWN need to be further clarified.

## MATERIALS AND METHODS

### Nematode

In August 2017 and December 2017, trees infected by PWNs were sampled in the same Chinese red pine forest in Fushun (N 41°58′, E 124°24′), Liaoning Province, northeastern China. Fushun belongs to the mid temperate zone, enjoying a continental monsoon climate. Summer is warm and rainy, but winter is very cold. The average annual temperature is 6.3°C, and the average winter temperature is −12.1°C. In the Chinese red pine forest, *Pinus tabulaeformis* can be classified into four categories based on the degree of PWN-associated damage. Seven Chinese red pines with trunk diameters of ∼12 cm that were normal in appearance, or had a small number of needles turning yellow, were selected, and 5–10 cm thick discs were taken from the upper, middle and lower parts of each pine. Using the Baermann funnel method, the PWNs were immediately isolated from the wood samples.

### Morphological characteristics

The isolated nematodes were observed and photographed using optical microscopy and stained with Oil Red O. Oil Red O staining was performed as outlined in Stamps's method, to observe the nematode lipid droplet areas (Stamps and Linit, 1995).

### Low temperature survival

The nematodes isolated from the wood samples collected in winter were placed in centrifuge tubes containing 1 ml of water to form a nematode suspension (5000 nematodes per ml). Then, 100 μl samples of the nematode suspensions, each containing approximately 500 PWNs were placed in Eppendorf tubes (four replicates) and transferred to the Low-temperature Reaction Bath.

The nematodes isolated from the wood samples collected in summer were placed in centrifuge tubes containing 1 ml of water to form a nematode suspension (5000 nematodes per ml). Then, 100 μl samples of the nematode suspensions, each containing approximately 500 PWNs, were placed in Eppendorf tubes (four replicates) and transferred to the Low-temperature Reaction Bath.

#### Frozen survival

The samples were controlled from 1°C to various temperatures (T_min_: 5, 0, −5, −10, −15°C) at 1°C min^−1^ and frozen by adding ice crystals. They were held at T_min_ for 12 h and then rewarmed to 1°C at 1°C min^−1^. After thawing, the samples were placed at room temperature for 24 h.

#### Unfrozen survival

The samples were cooled from 1°C to two minimum temperatures (T_min_: −5, −10) at 1°C min^−1^. They remained unfrozen at T_min_ for 12 h. They were then rewarmed to 1°C at 1°C min^−1^ and placed at room temperature for 24 h. Ultra-pure water was used to prepare the nematode suspensions to ensure they remained unfrozen. Survival was determined by counting the proportion of moving nematodes after a mechanical stimulus. Control samples consisted of 100 μl nematode suspension in Eppendorf tubes held at room temperature for the duration of the experiment.

### Gas chromatography for lipid

The nematodes isolated from the wood samples collected in winter and summer were independently added to centrifuge tubes containing 1 ml of water to form two nematode suspensions (25,000 nematodes per ml). Then, 100 μl samples of the nematode suspensions containing 2500 winter or summer forms of the PWN were independently placed in Eppendorf tubes. The samples were centrifuged, the dH_2_O supernatant removed and 20 μl C19:0 added as an internal standard. The mixtures were homogenized on ice. The homogenates were transferred to glass tubes and then incubated with 1 ml of methanol +2% H_2_SO_4_ for 1 h at 80°C. Once the samples cooled, 1 ml of hexane was added to each. Samples were oscillated, centrifuged at 1500× ***g*** for 3 min and the upper organic layers were extracted for GC analysis.

Samples were then analyzed on an Agilent 7890A GC (Agilent, USA) equipped with an AB FFAP column (30 m×250 μm×0.25 μm). A constant pressure of 15 psi was applied to the column. The oven temperature at the injection was 50°C. It was increased to 200°C at a rate of 10°C/min and held for 15 min. The flame ionization detector temperature was 270°C.

Each experiment was repeated at least three times. Average values and standard deviations were then calculated for each of the compounds in the experiments.

### Gas chromatography for sugars and polyols

Samples of 100 μl nematode suspensions, independently containing 5000 dispersal and propagative forms of the PWN, were placed in Eppendorf tubes. The samples were centrifuged, and the dH_2_O supernatant removed. Then, 400 μl of 80% (v/v) ethanol was added to the samples, and lactose was added as an internal standard. The mixtures were homogenized on ice for 1 min, centrifuged at 10,000× ***g*** for 5 min, and then, the supernatants were collected in vials. The supernatants were dried completely under a stream of nitrogen gas. The samples were dissolved in 0.2 ml dimethylformamide and converted to their trimethylsilyl derivatives by adding 100 μl N, O-bis (trimethylsilyl) trifluoroacetamide +1% trimethylchlorosilane. The vials were capped, incubated for 30 min at room temperature and then, centrifuged at 10,000× ***g*** for 5 min. The supernatants were collected for GC analysis.

Samples were then analyzed on an Agilent 7890A GC (Agilent) equipped with an HP-5 column (30 m×0.25 mm×0.25 μm). A constant pressure of 15 psi was applied to the column. The oven temperature at the injection was 120°C. It was increased to 280°C at a rate of 10°C/min and then held for 10 min. The flame ionization detector temperature was 270°C.

Each experiment was repeated at least three times. Average values and standard deviations were then calculated for each of the compounds in the experiments.

### Statistical analyses

All statistical analyses were calculated using SPSS v. 19.0 software. Probit analysis models were used to determine the temperature at which 50% of the nematodes were killed (S_50_). One-way ANOVAs were used to detect the effects of treatments, which were considered statistically significant at *P*<0.05.

## Supplementary Material

Supplementary information
