# Supplementary material for: Cold adaptive potential of pine wood nematodes overwintering in plant hosts
Source: Biol Open. 2019 Apr 25;8(5):bio041616. doi: 10.1242/bio.041616 (PMC6550080; doi:10.1242/bio.041616)
Supplement: Supplementary information [file biolopen-8-041616-s1.pdf]

Supplemental Material

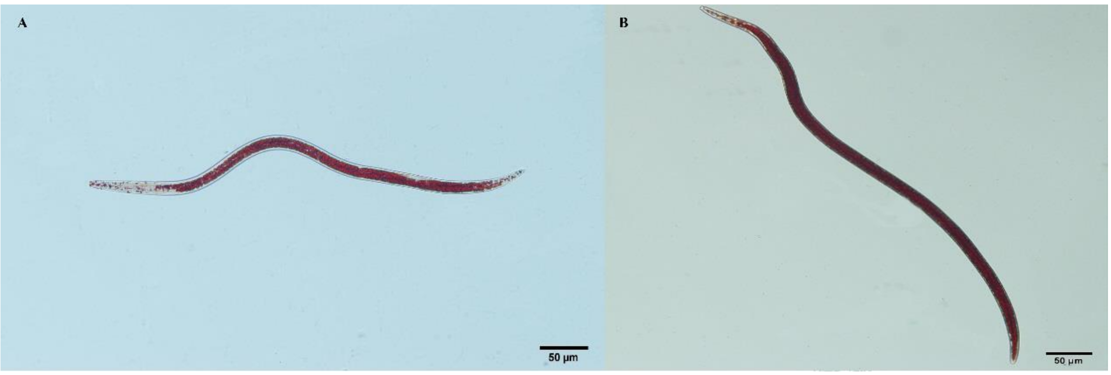

Figure S1 Photomicrographs of Oil Red O stained pine wood nematode propagative and dispersal larvae

A, propagative larvae; B, dispersal larvae

Biology Open • Supplementary information
